# Comparison of Spectral and Image Morphological Analysis for Egg Early Hatching Property Detection Based on Hyperspectral Imaging

**DOI:** 10.1371/journal.pone.0088659

**Published:** 2014-02-13

**Authors:** Wei Zhang, Leiqing Pan, Kang Tu, Qiang Zhang, Ming Liu

**Affiliations:** 1 College of Food Science and Technology, Nanjing Agricultural University, Nanjing, Jiangsu, PR China; 2 China National Research Institute of Food & Fermentation Industries, Beijing, PR China; Queensland Institute of Medical Research, Australia

## Abstract

The use of non-destructive methods to detect egg hatching properties could increase efficiency in commercial hatcheries by saving space, reducing costs, and ensuring hatching quality. For this purpose, a hyperspectral imaging system was built to detect embryo development and vitality using spectral and morphological information of hatching eggs. A total of 150 green shell eggs were used, and hyperspectral images were collected for every egg on day 0, 1, 2, 3 and 4 of incubation. After imaging, two analysis methods were developed to extract egg hatching characteristic. Firstly, hyperspectral images of samples were evaluated using Principal Component Analysis (PCA) and only one optimal band with 822 nm was selected for extracting spectral characteristics of hatching egg. Secondly, an image segmentation algorithm was applied to isolate the image morphologic characteristics of hatching egg. To investigate the applicability of spectral and image morphological analysis for detecting egg early hatching properties, Learning Vector Quantization neural network (LVQNN) was employed. The experimental results demonstrated that model using image morphological characteristics could achieve better accuracy and generalization than using spectral characteristic parameters, and the discrimination accuracy for eggs with embryo development were 97% at day 3, 100% at day 4. In addition, the recognition results for eggs with weak embryo development reached 81% at day 3, and 92% at day 4. This study suggested that image morphological analysis was a novel application of hyperspectral imaging technology to detect egg early hatching properties.

## Introduction

The incubation of chicken egg hatching takes about 21 days which is a time and energy-consuming process. Statistics show that egg embryo development rate was from 86% to 95%, which means every year there still are a large proportion of eggs cannot be hatched successfully. These non-hatchable eggs pose troubles to the industry because they take up space and energy, and have the potential to spread bacteria or molds, contaminate an entire hatching cabinet. Therefore, the development of an efficient, non-destructive, and accurate method for detecting egg hatching property will be advantageous to the industry.

In recent years, hyperspectral imaging method, which combines the advantages of imaging and spectroscopy to acquire spectral and image information simultaneously, has been proposed as a promising method for detecting a variety of agricultural products. Examples include detection quality of meat [1], [2], [3], injury in fruit and vegetable [4], [5], cracks in shell eggs [6].

Some scholars have used this technology in egg fertility and embryo development detection. For white shell egg, Liu et al. used a near-infrared hyperspectral imaging with the wavelength range between 900 and 1,700 nm, they obtained promising results for fertility detection, but classification results for early embryo development detection were low [7]. For brown shell egg, Lawrence et al. conducted a hyperspectral investigation for early embryo development with only 36 samples [8]. The similar experiment was taken by Smith et al., but the detection results were not good enough for industry (71% accuracy for day 0; 63% for day 1, 65% for day 2 and 83% for day 3) [9]. These research works provided good references and resources for dealing with various problems associated with detection of embryo development during early incubation. However, they applied relative small sample size, and focused only on the spectral characteristic of hatching egg which might be unstable, in addition, as mentioned above the recognition accuracy for embryo development with colored (brown) eggshell was not satisfied. The evolution of embryo development contained the changes of light transmission and morphologic characteristics, thus image morphological information of hyperspectral images might give a new revelation for detecting egg hatching property. In addition, to our knowledge no attempts have been made to detect the embryo vitality (e.g. weak embryo), which might be result in late embryonic mortality.

In non-destructive modeling of agricultural produces, neural networks can model nonlinear systems and have been widely used [10], [11]. There are different architectures of neural networks, and each has its own strengths and drawbacks, and good performance of a given architecture in a particular problem does not ensure similar results in a different problem. Learning vector quantization neural network (LVQNN) is a nearest-neighbor pattern classifier based on competitive learning [12] and in recent years, with the advantages of simple network and better pattern recognition, LVQNN have been widely applied for pattern classification, identification and pattern recognition analysis [13], [14].

The main objective of the present study was to investigate the applicability of spectral and image morphological analysis based on hyperspectral imaging for detecting embryo development and vitality of hatching eggs with green shell. The research was conducted through (1) set up a hyperspectral imaging system with a spectral region from 400 to 1000 nm to capture spectral and morphological information of hatching eggs; (2) determination of effective wavelengths for early embryo development and vitality detection based on Principal Component Analysis (PCA) method; (3) development of an algorithm to isolate image morphological of hatching eggs; (4) employment of LVQNN model to investigate the applicability of spectral and image morphological analysis for detecting embryo development and vitality during the first few days of incubation.

## Materials and Methods

### 1. Ethics Statement

This study was carried out in strict accordance with the animal protocol in the Guide for the Care and Use of Laboratory Animals. The protocol was approved by the Ethical Committee of Animal Experiments of Nanjing Agricultural University (Permit Number: SYXK (su) 2011–0036). The study did not involve endangered or protected species. All efforts were made to minimize animal suffering. Eggs were incubated in an incubator with a comfortable temperature and humidity, and the chick embryo sacrificed by a natural death.

### 2. Eggs and Sample Evolution

150 green shell eggs were freshly obtained from the Huang Mu Qiao hatchery in Nanjing, among which 90 eggs were immediately incubated in an incubator (HJ-TL, Haijiang incubation equipment manufacture Co., China) at the temperature of 38.5°C, relative humidity of 65%, and were turned automatically every two hours. On the incubation days of 0, 1, 2, 3, and 4, eggs were taken out for image capture by the hyperspectral imaging system in sequence and then immediately put back in the incubator. The rest of 60 eggs were first stored in a humidity chamber with the temperature of 15°C, relative humidity of 65% for 9 days, which would induce weak embryonic vitality [15], [16], and later they were taken the same approach as above.

After 5 days of incubation, eggs were broken out to determined embryo development and viability by referring to the characteristics table of embryonic development [17]. The embryo development of every egg was checked, and all the egg samples were divided into three groups: eggs with non developed embryo (N), eggs with embryo development (D) and eggs with weak developed embryo (W), in the rest of the paper, the egg samples were classified using neural network.

### 3. Hyperspectral Imaging System

**Figure 1:**
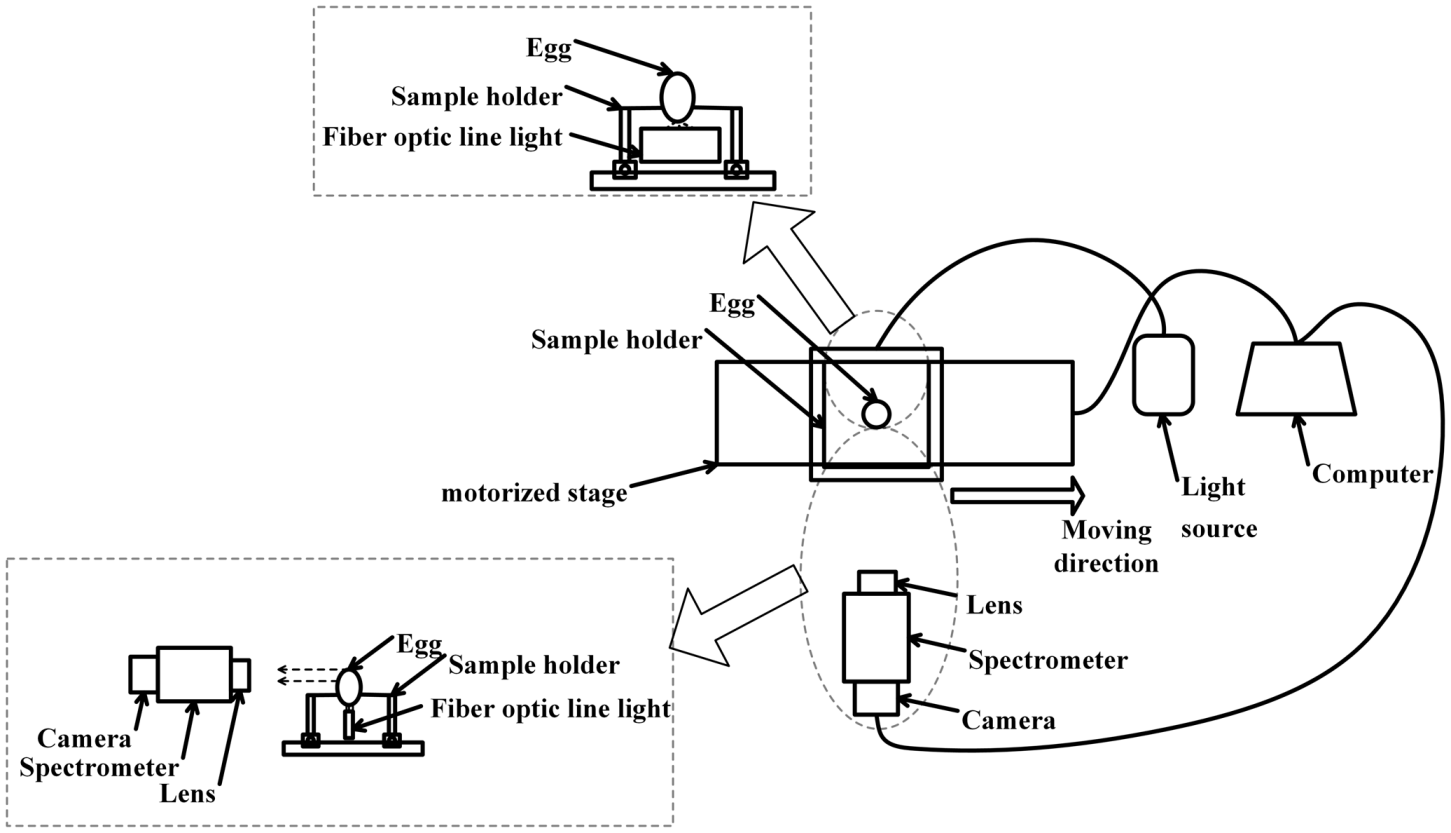
The schematic diagram of hyperspectral imaging system.

According to the research objective, spectral and image morphological information of egg was acquired, and a set of high spectral image acquisition system based on transmittance mode is shown in Fig. 1. This system mainly consists of a hyperspectral imaging unit, a DC tunable light source constituted by a 150 W halogen tungsten lamp controller (3900ER, Illumination Technologies Inc, USA), a sample holder station, an electronic control horizontal motorized stage (IRCP0076 of-ICOMB001, Isuzu, Taiwan, China), a computer (CPU E5800, 3.2 GHz, Memory 2 G, graphics 256 M GeForce GT240, Dell, USA) and the image acquisition software (Spectral Image, Isuzu, Taiwan, China).

Hyperspectral image unit was combined by a CCD camera (ICL-B1620, Imperx, USA), an imaging spectrometer (ImSpectorV10E, Specim, Finland) with a spectral resolution of 2.8 nm and a variable focal length lens. The effective wavelength of this system ranges from 400 to1000 nm. In order to avoid the interference of the external light source, the entire apparatus was placed in a black box.

### 4. Hyperspectral Image Acquisition and Correction

In order to acquire accurate data, some parameters need to be adjusted before the image acquisition such as camera exposure time, focal length, light intensity, image size and convey speed [18]. In this study, the parameters were determined as: the exposure time of 55 ms, the convey speed was adjusted to 2.5 mms^−1^, and the images were recorded with spatial dimension of 440 by 804 pixels. Thereafter, egg samples were vertically placed on the sample holder and conveyed to be scanned on line (Fig. 1). Thus, after images of all eggs were captured, a total of 750 images (150 egg samples×5 days) were obtained.

Due to the presence of the dark current in the camera and the impact of external factors, images contains certain noise [19], so that hyperspectral images were corrected with a white and a dark references [2]. The dark image (*R*_d_) was used to remove the effect of dark current of the camera when the light source was turned off and the camera lens was completely covered with its opaque cap. A Teflon white board was used to obtain white transmittance image (*R*_t_). The corrected relative image *R* was calculated according to formula (1). All the corrected images were then used as the basis for subsequent analysis to extract spectral information, image segmentation, effective wavelength selection, and detection of embryo development and vitality.
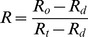
(1)where *R*_o_ is the original hyperspectral image for sample; *R*_d_ is the dark image (with 0% transmittance) and *R_t_* is the white transmittance image (with 99% transmittance).

### 5. Selection of Regions of Interest and Extraction of Spectral Characteristics

Hyperspectral imaging systems acquire abundant spatial information while collecting spectral information for each pixel in the image. It is an established procedure to select a representative region of interest (ROI) encompassed by pixels with similar signature for extracting spectral characteristics. On the other hand, gas exchanged through the pores at the surface of the eggshell during incubation, and the transmittance of spectra varied between pores and none-pore parts of the eggshell. Considering this, the ROI with the size of 1000 pixels was selected for each egg, and mean spectrum of ROI region from the Hyperspectral images was then calculated (i.e. the average of the transmission values of each pixel in the ROI in the current wavelength) over the spectral range of 400–1000 nm, and the peak value was produced at the highest transmission value of the mean spectrum. In total, 750 mean peak values were obtained from ROI of 150 hatching eggs for 5 days.

### 6. Image Segmentation and Morphologic Characteristics Extraction

**Figure 2:**
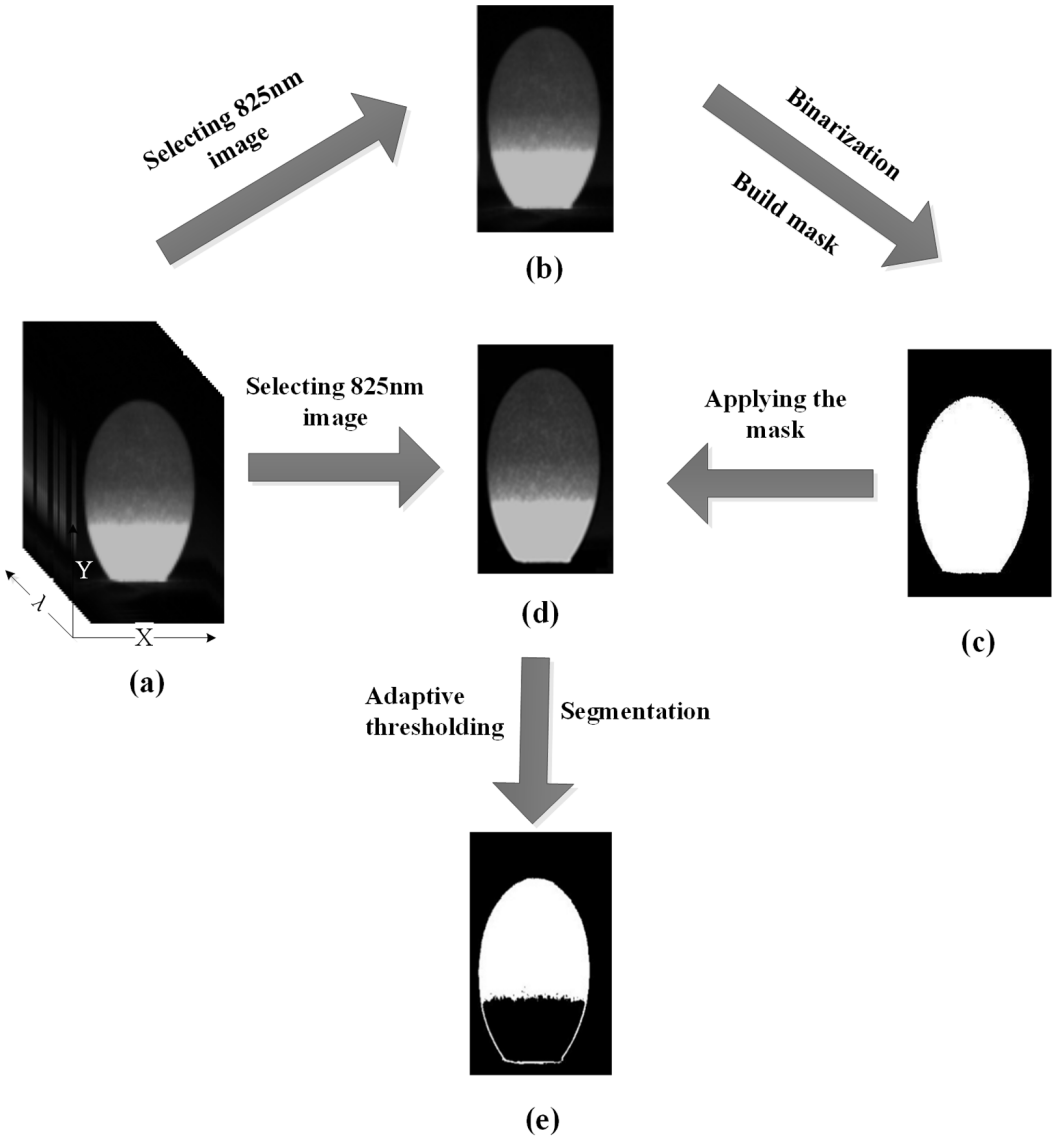
Flow chart of the key steps involved in embryo characteristics segmentation algorithm. (a) hyperspectral images with 440 spectral bands (b) image at 825 nm (c) binary mask for background removal (d) masked image after background removal (e) final binary image that contains only embryo.

The segmentation steps were developed to isolate the morphologic characteristics of embryo development in the hatching eggs with the aid of ENVI software as shown in Fig. 2. Firstly, the background was removed from the hatching egg image by applying masking. The image at the 825 nm band (Fig. 2b) was chosen for this task since the egg image appeared clear when compared with the background and could be easily segmented. A binary image was obtained based on the image at 825 nm using a thresholding value of 0.125, and this step produced a binary image called ‘Mask’ which contains only the egg (Fig. 2c). Secondly, the adaptive thresholding method was used to cut the embryo development morphologic from ‘Mask’ region of the image, and the embryo development morphologic characteristics was isolated to produce the final binary image which was shown in white pixels (1), and the background as well as the rest of ‘Mask’ region were shown in black pixels (0) (Fig. 2e).

### 7. Data Processing and Pattern Recognition

All image processing and analyzing were performed using the Environment for Visualizing Images software program (ENVI 4.7,Research System Inc., Boulder, CO, USA) and the experimental results of model evaluation were analyzed statistically using statistical toolbox of MATLAB 2009a (The MathWorks Inc., Natick, USA).

The hyperspectral imaging acquisition generates immense amount of spectral data, and extracting effective information from them is difficult. According to Wold et al., optimal wavelengths may be equally or more efficient than full wavelengths [20]. Therefore, it is important to find a proper data mining for optimum selection of most effective wavelengths without losing the important information from the corrected relative image. Principal component analysis (PCA) is widely used for dimensionality reduction and feature extraction [21], [22]. In our research, the ENVI software package was employed to extract optimal wavelengths from the full wavelength range for the efficient classification with the PCA method.

**Figure 3:**
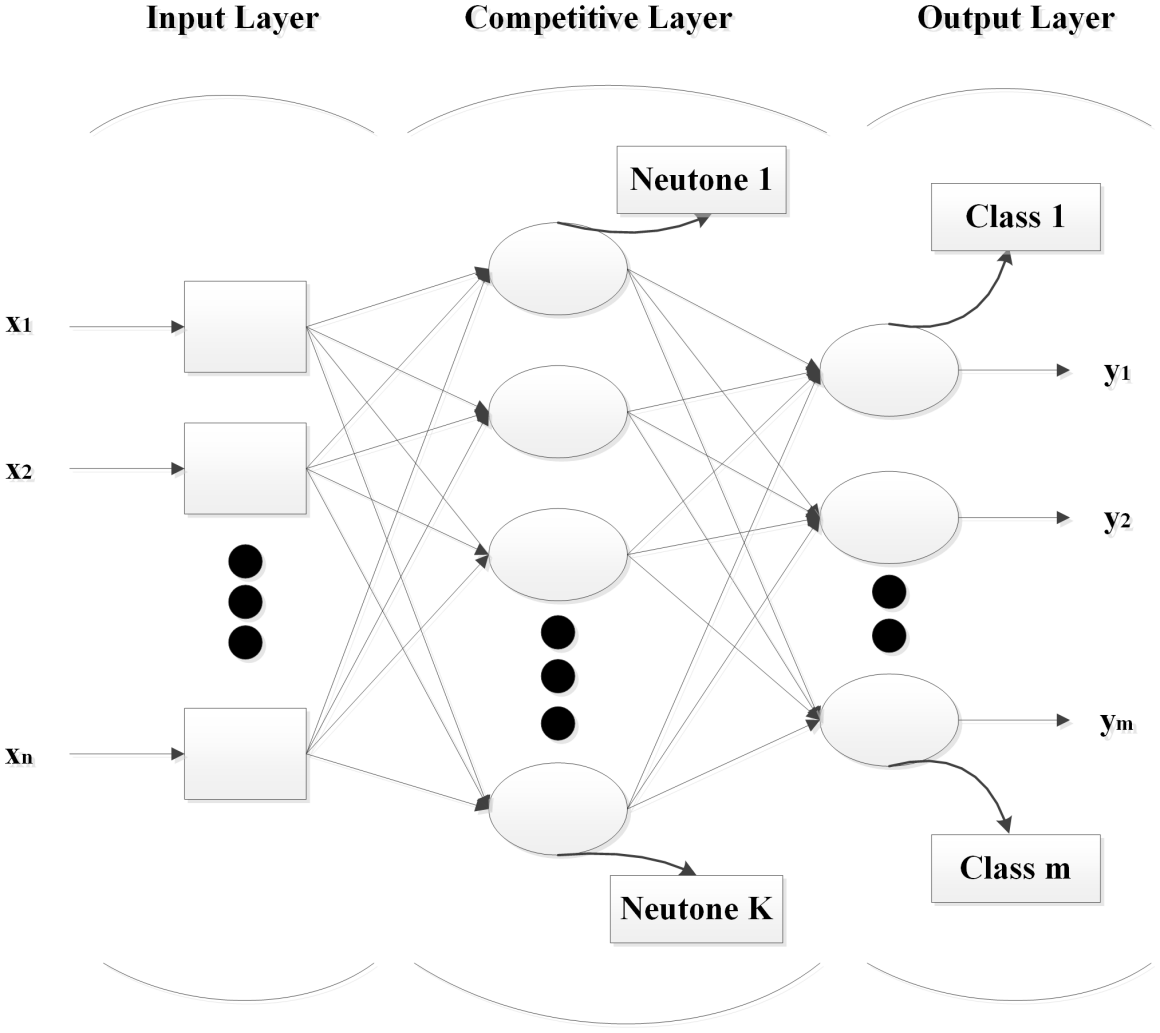
Structure under layers of the LVQ neural network.

In this study, the eggs were also analyzed by LVQNN. The LVQ neural network includes an input layer, a competitive layer which learns and performs the classification, and an output layer. The input layer contains one node for each input feature, and the output layer contains one node for each level of egg embryo development. Fig. 3 illustrates the structure of LVQ neural network. The egg samples of each group were separated as training set (two thirds of the samples) and testing set (one third of the samples) for the LVQNN model. To evaluate the training process and predict the results, the training accuracy were defined as the ratio of correctly classified number of training samples over the total number of training samples, and the testing accuracy were defined as the ratio of the correctly classified number of testing samples over the total number of testing samples, respectively.

Due to errors in the model repetitious test results for the same sample, LVQNN model test results of five times running were used for the ultimate experimental result. The coefficient of variance (cv) was to measure the variability of the model, calculated by the formula (2).
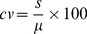
(2)where *s* is standard deviation; *μ* is mean value.

## Results and Discussion

### 1. Spectral Transmission Characteristics

**Figure 4:**
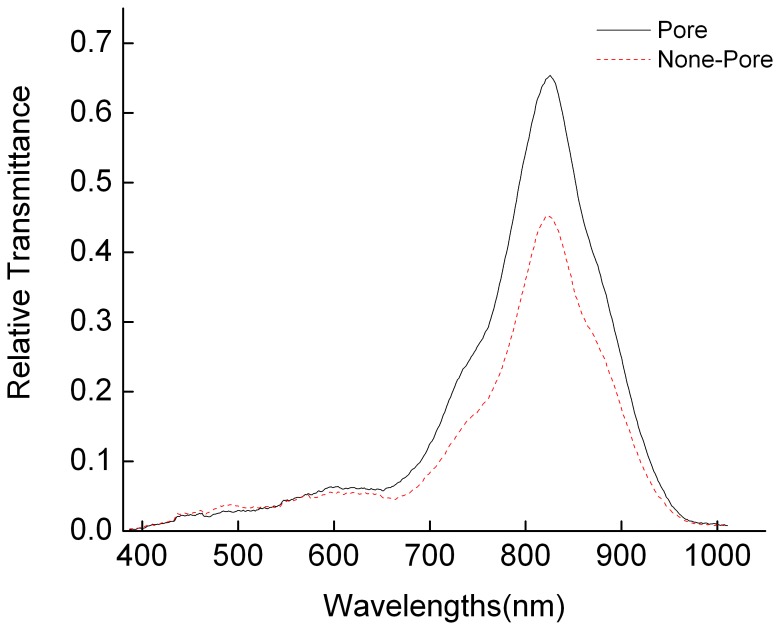
Typical transmittance spectra of different area of eggshell.

The typical relative transmittance spectra from pore and normal part (none-pore) of the same egg at day 4 are shown in Fig. 4. The transmittance spectra were close to 0 in the range of 400–650 and 950–1000 nm, which meant the signal-to-noise ratio was low and there existed no obvious spectral characteristic. In the range of 650–950 nm, the spectral curve first increased and then decreased, and the peak value occurred at the 825 nm, which indicated that the values of transmittance spectra around the peak could be used to detect eggs hatching property [7]. Usually, chicken embryo started the formation of red blood cells from day 2 on, which means the obvious absorption wavelength should be between 400 and 700 nm. Zhang et al. reported the characteristic bands of 571, 614, 661, 691 and 716 nm were the best wavelengths to detect chicken embryo development for white shelled eggs [23]. However, in our research, the peak value of transmittance spectra was found at 825 nm. A probable explanation is that there exist pigmentation with biliverdin and protoporphyrin in the green shell chicken [24], [25], which might influence spectral curve characteristics (the drift of spectral absorption peak).

**Figure 5:**
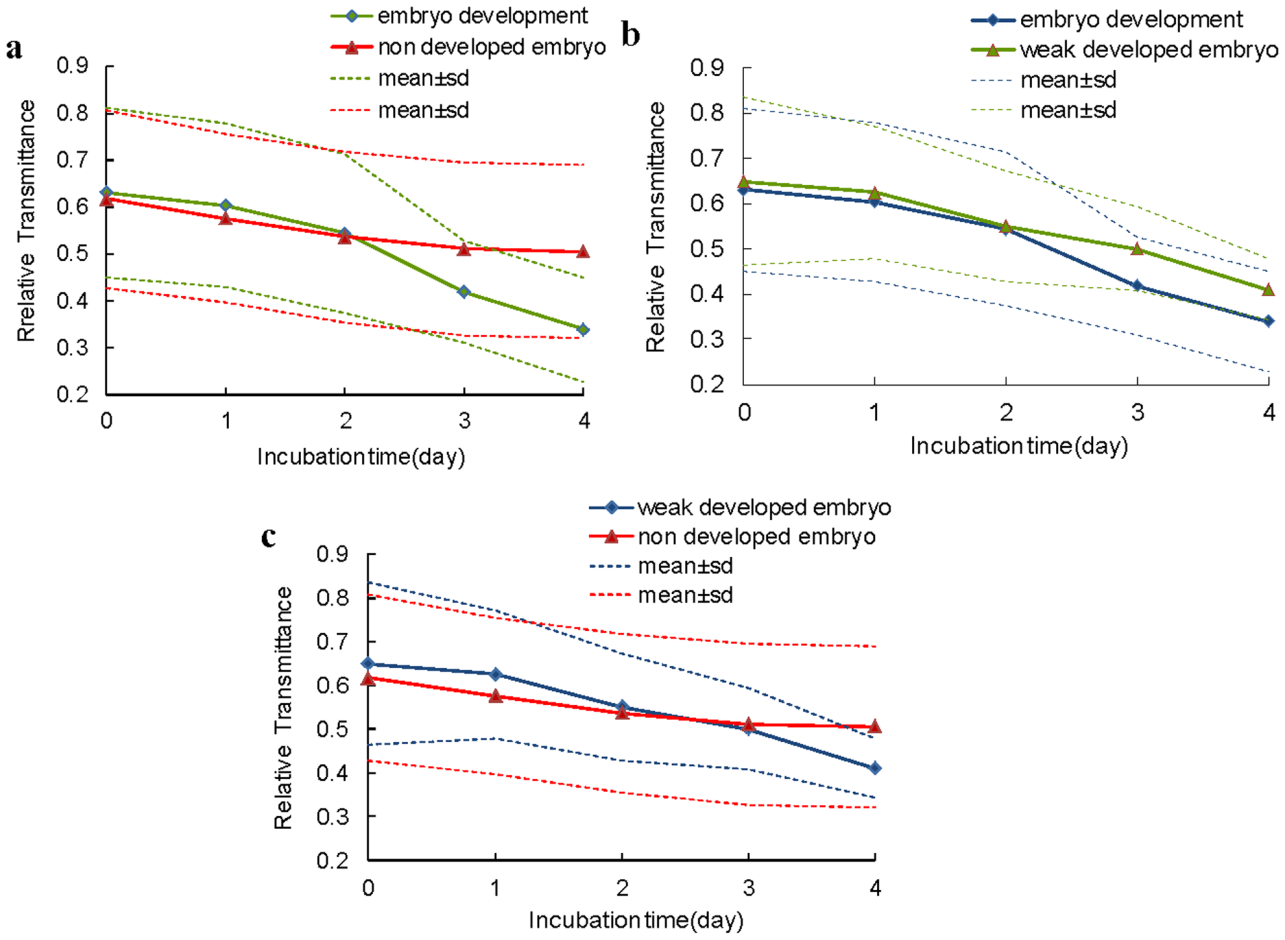
Evolution of mean peak value extracted from the ROI of the hyperspectral images of eggs in different groups.

The evolution of mean peak value of transmittance spectra from ROI for different groups of eggs is shown in Fig. 5. The mean peak values of eggs with non developed embryo (group N) kept decreased slowly during incubation. In contrast, the mean peak values of eggs with embryo development (group D) decreased significantly during incubation and dropped down more obvious at days 3 and 4 than days 1 and 2, which was similar to the report for the white shelled eggs [7]. The big difference of the mean peak value between eggs with non developed embryo and eggs with embryo development was observed since day 3 (Fig. 5a), which indicated that the two groups of eggs could be distinguished. However, this result was 1 day later than the white eggshell fertile detection [23]. This disparity could be explained by that the pigments in the green eggshell lowered the changes in light transmission during early embryo development [9]. The influence of eggshell was also existed in the eggs with weak developed embryo (group W), due to the weak embryonic vitality, the gap between eggs with weak developed embryo and eggs with non developed embryo was postponed to day 4 (Fig. 5b), and there was a difference between eggs with weak developed embryo and eggs with embryo development since day 3 (Fig. 5c). In addition, the mean peak value of different groups of eggs had the similar variation tendency at hatching days 0, 1 and 2, which indicated that these groups could not be separated from each other on these days.

### 2. Image Morphologic Characteristics of Embryo Development

**Figure 6:**
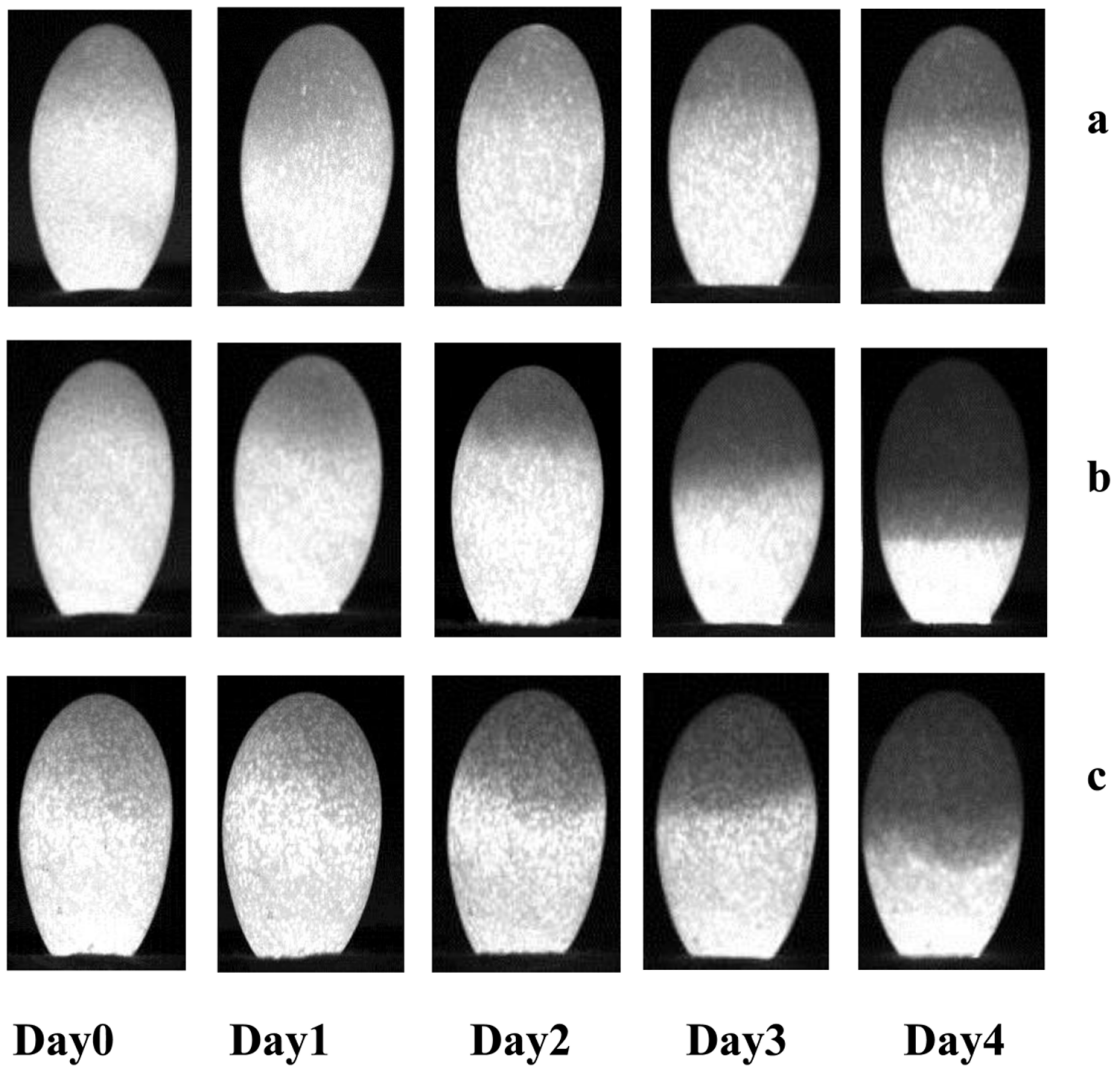
Gray level images illustrating sample presentation of the evolution of embryo development for eggs of different groups. a. egg with non developed embryo. b. egg with embryo development. c. egg with weak developed embryo.

The evolution of image morphologic characteristics of embryo development for eggs in different groups were randomly selected from the egg samples and shown in fig. 6. There were almost no changes in image morphologic characteristics of eggs with non developed embryo during the first four days of incubation (Fig. 6a, group N), which was logical when non embryo developed. In contrast, the eggs showed significant embryo development since day 3 if the egg belongs to the D group, and the phenomenon was more obvious at day 4 (Fig. 6b). For eggs with weak developed embryo (Fig. 6c, group W), the changes in embryo were slow on day 0, 1, 2, 3 and 4, and the changes were not obvious comparing to eggs with normal development of embryo. The phenomenon was due to the weak embryonic vitality and the slow embryo development. In addition, more obvious differences of morphologic characteristics of embryo development between the N, D and W groups of eggs were observed at day 4, which indicated that these groups could be clearly distinguished. These results were consistent with the spectral transmission characteristics discussed above.

### 3. Selection of Optimum Input Characteristic Parameters of LVQ Model

#### 3.1. Spectral characteristic parameter determination

**Figure 7:**
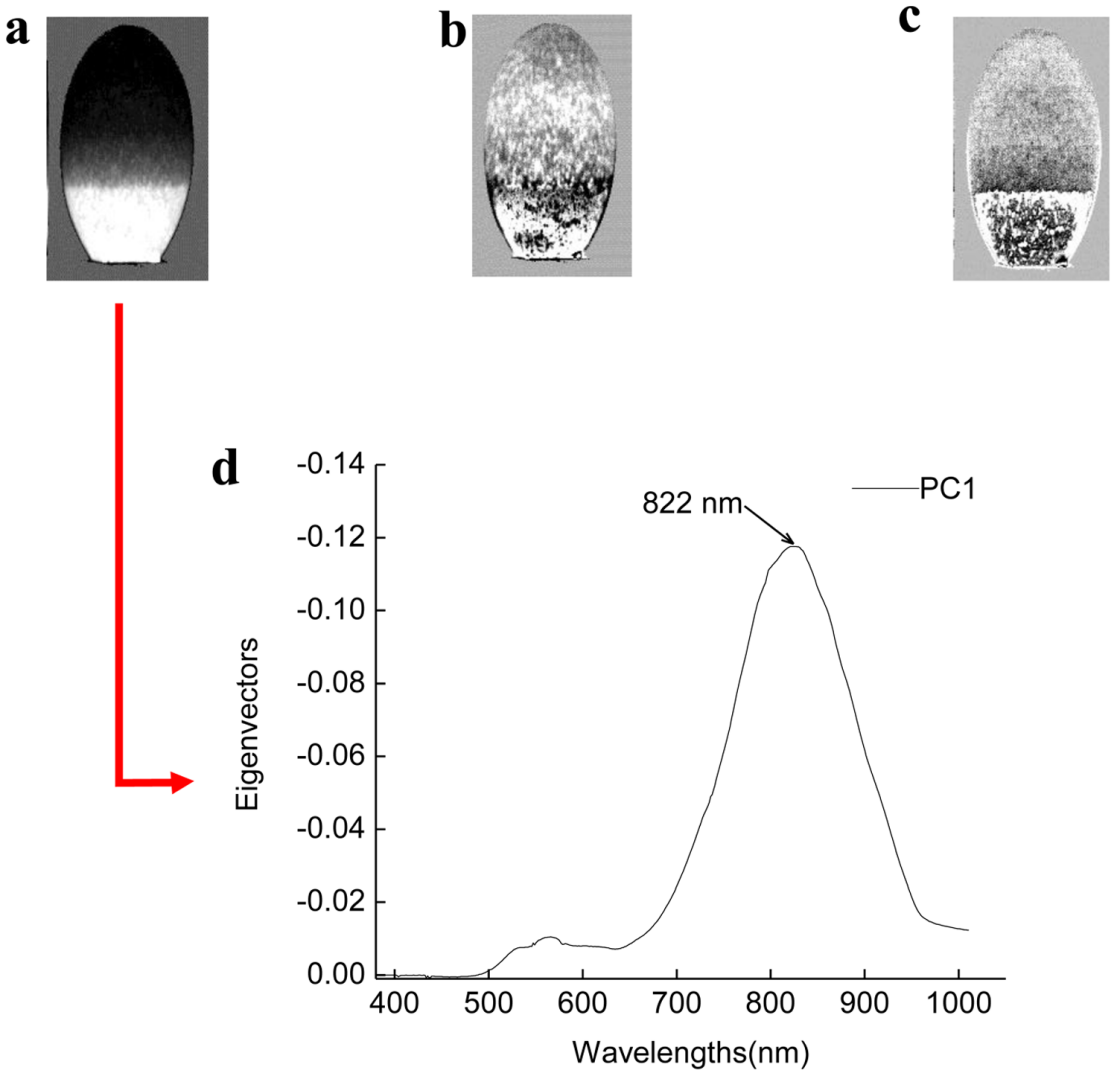
Principal component images based on the entire spectral region. (a-c) first three principle component (PC1, PC2 and PC3) images; (d) selection of optimal wavelengths from eigenvectors of PC1 image.

The egg sample images across the entire spectral region were analyzed by PCA. PC1, PC2 and PC3 images are shown in Fig. 7a–c, which are the first three PC images obtained by PCA. The variance contribution rate of PC1 image was 99.1%, which means PC1 image mostly explained the original spectral features. Thus, the wavelengths having high loading values at PC1 image were selected as effective wavelengths for extracting spectral characteristic. Fig. 7b shows the plot of wavelengths versus the loadings of PC1 image, It was decided to choose those wavelengths situated at the maximum or minimum, and only one wavelength (822 nm) was then selected. Finally, the mean peak transmission value of all pixels in the ROI at wavelength of 822 nm was chosen as the spectral characteristic parameters, calculated by the formula (3).
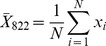
(3)where *x_i_* represented the peak transmission value at one point.

#### 3.2. Image characteristic parameters determination

**Figure 8:**
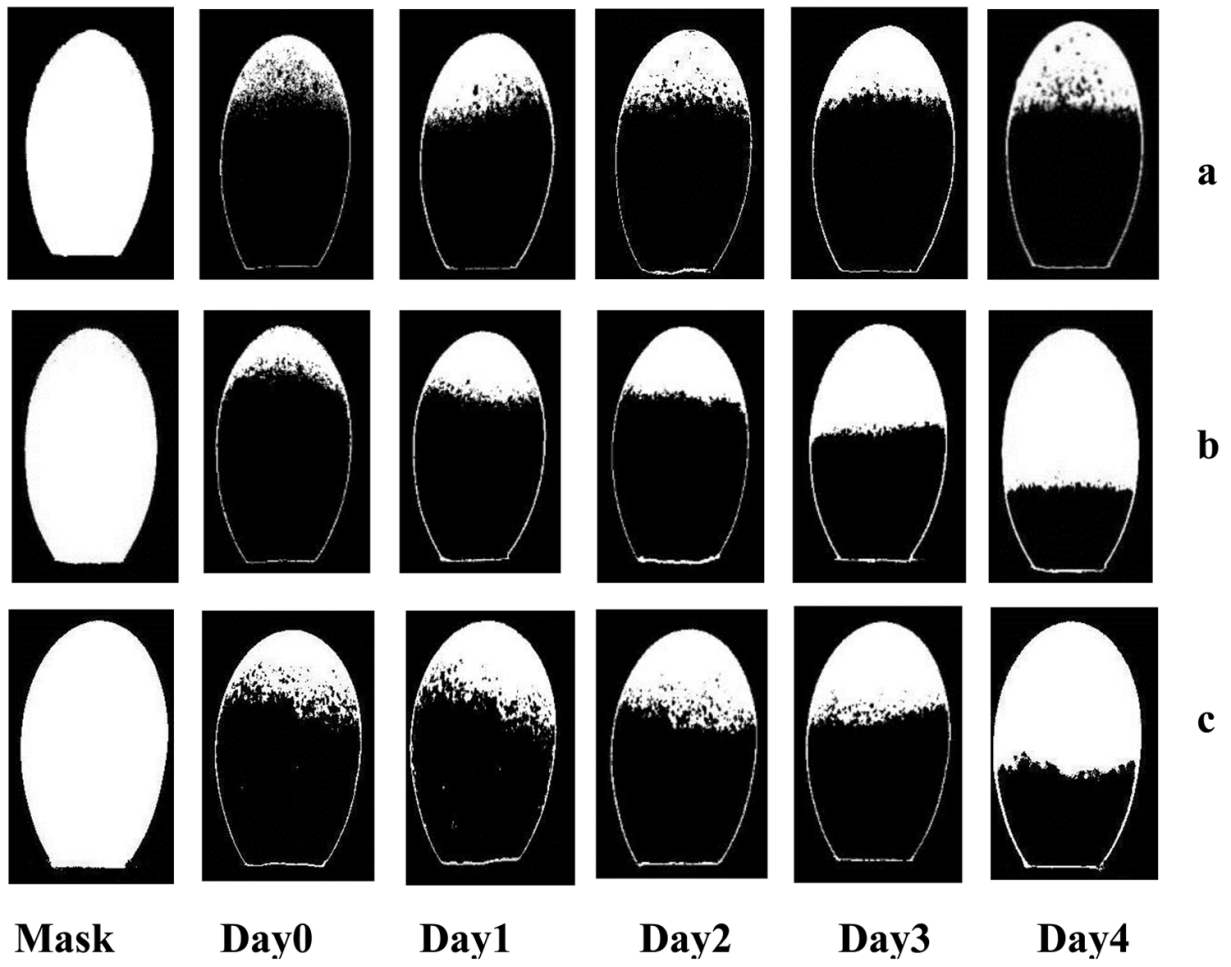
Binary images illustrating of image morphologic characteristics extraction analysis for eggs of different groups. a. egg with non developed embryo. b. egg with embryo development. c. egg with weak developed embryo.

An example of image morphologic characteristics extraction analysis performed by the proposed approach for three groups of eggs is shown in Fig. 8. At the left of each line was the ‘Mask’ region of the egg example. In the other binary images, the white regions represented morphologic characteristic of embryo development isolated from the three different groups of eggs, showing the effectiveness of the proposed segmentation algorithm for image morphologic characteristics extraction.

The morphologic characteristics of embryo development could be considered as the characteristic parameters. In order to avoid the influence of egg size, morphologic characteristic rate *W* was chosen as the characteristic parameter. The morphologic characteristic rate formula was described as follows:
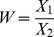
(4)where *X*_1_ is the pixel number of segmented binary image of morphologic characteristic; *X*_2_ is the pixel number of mask region image.

### 4. LVQNN Modeling Method

#### 4.1. Determination of the best neural network model parameters

Model parameter search played a crucial role in the performance of LVQNN. The parameters included number of neuron in the input layer, competitive layer, output layer, and the learning factor. After repeated tests, the optimum parameters were determined. The number of neuron in input layer was 1, because only one characteristic parameter (spectral characteristic parameter or image characteristic parameter) was chosen as the input variable at one time running; the competitive layer was determined as 10 calculated by the formula (5), and the number of neuron in output layer was determined as 2, because eggs with different embryo development were encoded as (1, 0) and (0, 1). The rest parameters were as follows: learning speed was set at 0.1, and training error was set at 0.01, and learnlv2 has been adopted as learning algorithm.
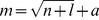
(5)where *m* is competitive layer number, *n* is input layer number, *l* is output layer number, *a* is an integer from 1 to 10.

#### 4.2. Hatching egg classification by LVQNN

A multilayer LVQNN was developed to investigate the applicability of spectral and image morphological analysis to discriminate eggs with non developed embryo from eggs with embryo development (N and/or D), and eggs with embryo development from eggs with weak developed embryo (D and/or W).

Table 1 shows the performance of ANN Model using spectral characteristic parameter 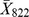 for classification of the training and the testing sets of egg samples. The ANN model classification results for eggs with different embryo development were low at Day 0, 1, 2 and 3 (e.g. N/D 77%, D/W 80% at Day3), the possible reason was that the spectral characteristics of early embryo development were influenced by pigmentation in green eggshell which was embodied in the large standard deviation of the mean peak value for the eggs (as shown in Fig. 5). The discrimination results improved at Day 4, and the classification result between N/D of testing set reached 83%, and the D/W was 82%. The results showed that the influence of eggshell was relevant to the development of embryo, and with the embryo development, the influence of eggshell became smaller. The spectral transmission characteristics could be used to detect egg hatching property with green shell, especially for incubation day 4.

**Table 1:** Recognition results of training and testing sets of egg samples by LVQ neural network based on spectral characteristic parameter.

Incubationtime/d	Non developed embryo/Embryo development(N/D)/%				Embryo development/Weak developed embryo (D/W)/%			
	Modelingaccuracy	cv	Predictionaccuracy	cv	Modelingaccuracy	cv	Predictionaccuracy	cv
Day 0	73±5	7	65±2	3	67±7	10	69±3	4
Day 1	74±4	5	63±2	3	72±5	7	72±5	7
Day 2	72±4	6	60±4	7	73±2	3	66±2	3
Day 3	81±5	6	77±6	8	83±2	2	80±4	5
Day 4	85±3	4	83±2	2	86±3	3	82±4	5

Table 2 shows the classification results of ANN model using image characteristic parameter *W*. The classification accuracies for testing set were low at Day 0, 1, 2 (e.g. N/D for 76%, D/W for 70% at Day2). However, the results improved since Day 3, the N/D classification results were 97% at Day3, 100% at Day4, and classification results of D/W were 81% at Day3, 92% at Day4.

**Table 2:** Recognition results of training and testing sets of egg samples by LVQ neural network based on image characteristic parameter.

Incubationtime/d	Non developed embryo/Embryo development (N/D)/%				Embryo development/Weak developed embryo (D/W)/%			
	Modelingaccuracy	cv	Predictionaccuracy	Cv	Modelingaccuracy	cv	Predictionaccuracy	cv
Day 0	74±3	4	72±2	3	73±5	7	73±4	5
Day 1	79±4	5	70±3	4	74±4	5	71±3	4
Day 2	82±1	1	76±2	3	76±3	4	70±3	4
Day 3	99±0	0	97±0	0	83±2	2	81±2	2
Day 4	100±0	0	100±0	0	93±1	1	92±2	2

Comparing the results listed in Table 1 and Table 2, model prediction accuracy using spectral characteristic parameter and image characteristic parameter were relatively low at Days 0, 1, 2, which implied that embryo development was not obvious (as shown in Fig. 6). The result was consistent with the fact that blood formation starts in the developing embryo from day 2 [26]. Since Day 3, classification accuracy by image characteristic parameter were better than by spectral characteristic parameter, such as the classification results for N/D at Day 3 were 97% vs. 77% and Day 4 100% vs. 83%, which implied that embryo development could be clearly detected. The results obtained here were better than those results of brown shell eggs reported by Smith et al. (2008) (72% vs. 71% for day 0; 70% vs. 63% for day 1, 76% vs. 65% for day 2 and 97% vs. 83% for day 3), and were also better than withe shell eggs for early embryo development detection studied by Liu et al. (2012) (76% vs. 74% for day 2. 97% vs. 82% for day 3, and 100% vs. 84% at day 4). In addition, the discrimination for eggs with weak developed embryo (D/W) reached 81% at day 3, 92% at day 4, which meant embryo activity could be detected by morphological characteristics. On the other hand, the *cv* of model classification results established by image parameters were much lower than by spectral transmission parameters at Day 3 (e.g. N/D 0 vs. 8), Day 4(e.g. N/D 0 vs. 2), and the low *cv* mean good generalization. These results suggested that the image morphological analysis was more suitable for detecting embryo development and vitality than spectral analysis for green shell egg. The probable reason was that pigments in the green eggshell lowered transmission of light, which would conceal the spectral characteristic of embryo development. Furthermore, pigmentation varying between pore and non-pore parts of eggshell may lead to the instability in spectral transmission characteristics. In contrast, image morphologic characteristics of embryo development were less affected by the eggshell.

## Conclusions

In this investigation, a special hyperspectral imaging system was built to detect embryo development and vitality using spectral and morphological information of hatching eggs. The high spectral dimensionality of transmission images data were reduced to few optimal wavelengths by PCA and one wavelength (822 nm) was selected instead of the full wavelength range for the detection of eggs hatching properties, and an image segmentation algorithm was applied to isolate the embryo development morphologic characteristics of hatching eggs.

The embryo development was not obvious at Day 0, 1, 2, and could not be detected correctly. The embryo showed significant development since day 3, and the changes became more obvious at day 4. However, embryo development was slow for eggs with weak developed embryo. The model test results showed that LVQNN using image characteristic parameter could achieve better prediction accuracy and generalization than using spectral characteristic parameter. The relatively better identification results between eggs with non developed embryo and eggs with embryo development were 97% at day 3, 100% at day 4, which implied that embryo development could be clearly detected. In addition, the discrimination for eggs with weak developed embryo (D/W) reached 81% at day 3, 92% at day 4, which meant embryo activity could be detected by morphological information.

The experimental results demonstrated that image morphological analysis of hyperspectral imaging system was a new method for egg hatching properties detection, which improved the detection accuracy for hatching eggs with colored shell, and had the ability to detect embryo activity.
